# Supplementary material for: Mapping current and future thermal limits to suitability for malaria transmission by the invasive mosquito Anopheles stephensi
Source: Malar J. 2023 Mar 21;22:104. doi: 10.1186/s12936-023-04531-4 (PMC10029218; doi:10.1186/s12936-023-04531-4)
Supplement: Supplementary file 1 — Additional file 1. Table S1. Top 10 Global Burden of Disease defined regional increase in people at risk (PAR) for year-round transmission suitability of P. falciparum by An. stephensi in 2050, under RCP 4.5 (SSP2 population projection) and RCP 8.5 (SSP5 population projection) future climate scenarios, averaged across four general circulation models (GCMs) as described in the main methods. Global increase is the sum of all gains in PAR increases across all GBD regions. Table S2.Top 10 Global Burden of Disease defined regional increase in people at risk (PAR) for year-round transmission suitability of P. vivax by An. stephensi in 2050, under RCP 4.5 (SSP2 population projection) and RCP 8.5 (SSP5 population projection) future climate scenarios, averaged across four general circulation models (GCMs) as described in the main methods. Global increase is the sum of all gains in PAR increases across all GBD regions. Table S3.Top 10 Global Burden of Disease defined regional increase in people at risk (PAR) for one or months transmission suitability of P. falciparum by An. stephensi in 2050, under RCP 4.5 (SSP2 population projection) and RCP 8.5 (SSP5 population projection) future climate scenarios, averaged across four general circulation models (GCMs) as described in the main methods. Global increase is the sum of all gains in PAR increases across all GBD regions. Table S4.Top 10 Global Burden of Disease defined regional increase in people at risk (PAR) for one or months transmission suitability of P. vivax by An. stephensi in 2050, under RCP 4.5 (SSP2 population projection) and RCP 8.5 (SSP5 population projection) future climate scenarios, averaged across four general circulation models (GCMs) as described in the main methods. Global increase is the sum of all gains in PAR increases across all GBD regions. [file 12936_2023_4531_MOESM1_ESM.docx]

Table S1. Top 10 Global Burden of Disease defined regional increase in people at risk (PAR) for year-round transmission suitability of *P. falciparum* by *An. stephensi* in 2050, under RCP 4.5 (SSP2 population projection) and RCP 8.5 (SSP5 population projection) future climate scenarios, averaged across four general circulation models (GCMs) as described in the main methods. Global increase is the sum of all gains in PAR increases across all GBD regions.

|  | **RCP 4.5** | | **RCP 8.5** | |
| --- | --- | --- | --- | --- |
|  | **GBD Region** | **PAR increase** | **GBD Region** | **PAR increase** |
| 1 | Sub-Saharan Africa (East) | 280,424,638 | Sub-Saharan Africa (East) | 189,762,004 |
| 2 | Sub-Saharan Africa (West) | 241,337,187 | Sub-Saharan Africa (West) | 119,410,162 |
| 3 | Asia (South) | 180,601,812 | Asia (East) | 42,024,538 |
| 4 | Sub-Saharan Africa (Central) | 56,637,965 | Sub-Saharan Africa (Central) | 35,356,077 |
| 5 | Latin America (Central) | 37,961,942 | Latin America (Central) | 23,650,404 |
| 6 | Asia (East) | 19,424,568 | North Africa & Middle East | 14,269,443 |
| 7 | North Africa & Middle East | 12,021,409 | Sub-Saharan Africa (Southern) | 13,103,271 |
| 8 | Sub-Saharan Africa (Southern) | 10,809,428 | North America (High Income) | 7,915,001 |
| 9 | Latin America (Tropical) | 9,282,480 | Latin America (Tropical) | 7,490,856 |
| 10 | Asia (Southeast) | 8,582,577 | Latin America (Southern) | 2,378,582 |
|  | **Global increase** | **871,809,779** |  | **460,335,572** |

Table S2. Top 10 Global Burden of Disease defined regional increase in people at risk (PAR) for year-round transmission suitability of *P. vivax* by *An. stephensi* in 2050, under RCP 4.5 (SSP2 population projection) and RCP 8.5 (SSP5 population projection) future climate scenarios, averaged across four general circulation models (GCMs) as described in the main methods. Global increase is the sum of all gains in PAR increases across all GBD regions.

|  | **RCP 4.5** | | **RCP 8.5** | |
| --- | --- | --- | --- | --- |
|  | **GBD Region** | **PAR increase** | **GBD Region** | **PAR increase** |
| 1 | Sub-Saharan Africa (East) | 225,798,598 | Sub-Saharan Africa (East) | 150,305,912 |
| 2 | Sub-Saharan Africa (Central) | 61,778,483 | Sub-Saharan Africa (Central) | 39,958,616 |
| 3 | Latin America (Central) | 17,400,921 | Sub-Saharan Africa (Southern) | 11,597,963 |
| 4 | Asia (East) | 10,755,605 | Asia (East) | 6,892,290 |
| 5 | Sub-Saharan Africa (Southern) | 7,401,600 | North America (High Income) | 2,612,318 |
| 6 | Latin America (Andean) | 3,751,711 | Latin America (Southern) | 2,293,282 |
| 7 | Latin America (Tropical) | 3,517,781 | Latin America (Andean) | 1,553,281 |
| 8 | North Africa & Middle East | 2,949,475 | Oceania | 1,232,957 |
| 9 | Latin America (Southern) | 2,311,302 | Australasia | 695,981 |
| 10 | North America (High Income) | 1,945,290 | North Africa & Middle East | 107,066 |
|  | **Global increase** | **339,670,521** |  | **217,249,667** |

Table S3. Top 10 Global Burden of Disease defined regional increase in people at risk (PAR) for one or months transmission suitability of *P. falciparum* by *An. stephensi* in 2050, under RCP 4.5 (SSP2 population projection) and RCP 8.5 (SSP5 population projection) future climate scenarios, averaged across four general circulation models (GCMs) as described in the main methods. Global increase is the sum of all gains in PAR increases across all GBD regions.

|  | **RCP 4.5** | | **RCP 8.5** | |
| --- | --- | --- | --- | --- |
|  | **GBD Region** | **PAR increase** | **GBD Region** | **PAR increase** |
| 1 | Asia (South) | 465,096,731 | Asia (South) | 187,123,365 |
| 2 | Sub-Saharan Africa (East) | 283,814,459 | Sub-Saharan Africa (East) | 171,277,189 |
| 3 | Sub-Saharan Africa (West) | 271,935,016 | Sub-Saharan Africa (West) | 165,798,742 |
| 4 | North Africa & Middle East | 154,006,142 | Europe (Western) | 152,051,451 |
| 5 | Europe (Western) | 75,893,897 | North America (High Income) | 147,947,883 |
| 6 | North America (High Income) | 67,415,234 | North Africa & Middle East | 87,744,795 |
| 7 | Asia (Southeast) | 50,393,023 | Sub-Saharan Africa (Central) | 25,042,405 |
| 8 | Sub-Saharan Africa (Central) | 48,162,272 | Australasia | 23,093,746 |
| 9 | Latin America (Central) | 44,930,762 | Latin America (Central) | 3,121,109 |
| 10 | Australasia | 14,947,865 | Sub-Saharan Africa (Southern) | 2,914,570 |
|  | **Global increase** | **1,516,731,989** |  | **968,115,736** |

Table S4. Top 10 Global Burden of Disease defined regional increase in people at risk (PAR) for one or months transmission suitability of *P. vivax* by *An. stephensi* in 2050, under RCP 4.5 (SSP2 population projection) and RCP 8.5 (SSP5 population projection) future climate scenarios, averaged across four general circulation models (GCMs) as described in the main methods. Global increase is the sum of all gains in PAR increases across all GBD regions.

|  | **RCP 4.5** | | **RCP 8.5** | |
| --- | --- | --- | --- | --- |
|  | **GBD Region** | **PAR increase** | **GBD Region** | **PAR increase** |
| 1 | Asia (South) | 465,270,699 | Asia (South) | 187,452,995 |
| 2 | Sub-Saharan Africa (East) | 287,838,551 | Europe (Western) | 180,670,956 |
| 3 | Sub-Saharan Africa (West) | 271,933,624 | Sub-Saharan Africa (East) | 175,513,403 |
| 4 | North Africa & Middle East | 154,326,951 | Sub-Saharan Africa (West) | 165,797,409 |
| 5 | Europe (Western) | 104,894,102 | North America (High Income) | 150,498,663 |
| 6 | North America (High Income) | 69,688,688 | North Africa & Middle East | 88,097,243 |
| 7 | Asia (Southeast) | 50,497,397 | Sub-Saharan Africa (Central) | 25,400,517 |
| 8 | Sub-Saharan Africa (Central) | 48,520,385 | Australasia | 23,311,798 |
| 9 | Latin America (Central) | 43,071,846 | Latin America (Central) | 6,272,702 |
| 10 | Australasia | 15,142,386 | Sub-Saharan Africa (Southern) | 2,926,014 |
|  | **Global increase** | **1,554,262,423** |  | **1,011,067,047** |
